# Recurrent Thoracic Intradural Extramedullary Calcifying Pseudoneoplasm

**DOI:** 10.1155/crip/6088106

**Published:** 2026-10-14

**Authors:** Masayuki Shintaku, Takeshi Matsui, Takahiro Fukuzaki, Masayuki Ishihara, Katsunori Uchida, Koji Tsuta

**Affiliations:** ^1^ Department of Pathology Kansai Medical University Hospital Hirakata Japan kmu.ac.jp; ^2^ Department of Orthopedics Kansai Medical University Hospital Hirakata Japan kmu.ac.jp

**Keywords:** calcifying pseudoneoplasm, meningothelial hyperplasia, pathogenesis, spinal cord, tumoral calcinosis

## Abstract

A case of calcifying pseudoneoplasm of the neuraxis (CAPNON) that arose as an intradural mass of the thoracic spinal cord of a 53‐year‐old man, a motorcycle mechanic, is reported. The mass showed regrowth after the subtotal removal and was resected again 8 years later. Both primary and regrowth lesions consisted of calcified round nodules of the myxochondroid matrix rimmed by polygonal or short spindle cells. Fibrous tissue showing chronic inflammatory cell infiltration intervened between the nodules. Lamellar or woven bone tissue was also observed. In the regrowth lesion, the myxochondroid matrix was reduced in amount and partly replaced by fibrous tissue. Cells rimming the myxochondroid matrix were immunoreactive to epithelial membrane antigen and somatostatin receptor Type 2A, supporting the meningothelial origin. The pathogenesis of CAPNON remains obscure, but it is a nonneoplastic, probably reactive disorder to some unknown causes, and its close histopathological resemblance to tumoral calcinosis of somatic soft tissue suggests that both disorders share a common pathogenesis. Meningothelial cells surrounding myxochondroid nodules in the present lesions probably represent reactive meningothelial hyperplasia and reflect the locoregional specificity of the lesions. In the present case, some chronological changes of histopathological findings could be observed by comparing the specimens of two operations. Occupational mechanical overload to the vertebral column for a long term might have contributed to the pathogenesis of CAPNON.

## 1. Introduction

Calcifying pseudoneoplasm of the neuraxis (CAPNON) is a rare, calcifying tumefactive disorder in which lesions arise along the neural axis and simulate neoplasms both clinically and pathologically [1–4]. The lesions frequently affect the spine or spinal cord and form an intradural, epidural, or, rarely, intraosseous mass. CAPNON is considered a nonneoplastic, reactive disorder and is usually treated by simple surgical excision, but postoperative regrowth has been recorded [2, 5, 6]. Histopathologically, CAPNON lesions consist of two tissue components: (1) the calcified myxochondroid matrix rimmed by polygonal or short spindle cells, forming “palisading granuloma”–like, large round nodules and (2) lamellar or woven bone tissue [2, 4]. The etiology and pathogenesis of CAPNON remain unknown, although the involvement of meningeal cells in the pathogenesis has been suggested by some investigators [1, 3, 4].

A case of CAPNON that arose as an intradural extramedullary mass of the thoracic cord and developed regrowth after subtotal resection is reported. The specimen obtained by the second operation showed some interesting chronological alterations of histopathological findings. The pathogenesis of CAPNON is discussed on the basis of the clinicopathological findings, with special reference to its histopathological resemblance to tumoral calcinosis of somatic soft tissue [7–10].

## 2. Case Presentation

### 2.1. Clinical History

The patient, a 53‐year‐old man who had worked more than 10 years as a motorcycle mechanic, was referred to our hospital for persistent lumbago and bilateral thigh pain. Lumbar disc herniation at the levels of L4/L5 and L5/S1 had been diagnosed and treated by analgesics for approximately 10 years. Magnetic resonance imaging (MRI) and computed tomography (CT) myelography on admission demonstrated an intradural extramedullary mass lesion on the left side of the spinal canal at the level of Th11/12 (Figure 1A,B). The mass, measuring about 20 mm in the craniocaudal direction, was partly calcified and compressed the spinal cord to the right side. It was homogeneously enhanced by the contrast medium and showed a dumbbell‐shaped extension through the intervertebral foramina. Lumbar disc herniation of mild degree was found at the L4/L5 and L5/S1 levels, but no other degenerative abnormalities were found in the vertebral column and other joints or ligaments. At operation, the partly calcified mass, which was attached to the spinal nerve roots, was subtotally resected.

**Figure 1:**
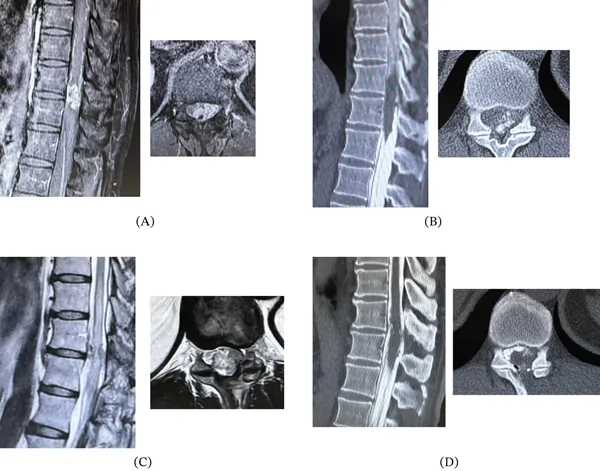
Neuroradiological findings. (A) A homogeneously enhanced tumorous lesion is found in the subdural extramedullary location at the level of T11/T12. (B) The contrast medium is blocked at the level from T11 to L1. The mass lesion is partly calcified. (C) The regrowth lesion is found at the same level as the primary lesion and extends further from the level of T9 to L1. (D) The contrast medium is blocked at the level from T11 to L1. The regrowth lesion is partly calcified. (A) MRI, T1‐weighted image with contrast enhancement. (B,D) CT myelography. (C) MRI, T2‐weighted image.

Postoperatively, the lumbago improved, and the patient returned to his work, although muscle weakness, numbness, and hypesthesia of the left lower extremities remained. However, at follow‐up 2 years later, regrowth of the mass was suspected on the radiological examination. Because the subjective symptoms remained mild, careful observation was chosen. The mass increased in size very slowly and was resected 8 years after the first operation (at the age of 61 years). The mass extended from the T9/T10 to T12/L1 levels (Figure 1C,D), and many small, partly calcified nodules were found to be disseminated in the intradural space of the caudal side. They were removed as far as possible, and then the patient was put in a rehabilitation program. He is able to walk with assistance of a cane 7 months after operation, although mild pain and hypesthesia of the left thigh still remain. Throughout the whole clinical course, serum calcium levels and renal function tests were within normal ranges. Serum phosphate, parathyroid hormone, and vitamin D were not examined, and no symptoms or signs suggestive of any metabolic disturbances were noted.

### 2.2. Pathological Findings

#### 2.2.1. Histopathology

The specimens obtained by both operations consisted of aggregates of many small pieces of whitish, gritty, friable tissue that showed approximately the same histopathological features. They consisted of two tissue components: (1) the hypocellular, calcified myxochondroid matrix and (2) lamellar or woven bone tissue. The myxochondroid matrix formed discrete or confluent, large round nodules and, being rimmed by polygonal or short spindle cells, showed a “palisading granuloma”–like appearance (Figure 2A). Similar polygonal or short spindle cells were also occasionally seen within the matrix. The matrix was palely basophilic and Alcian Blue–positive. It showed deposition of calcium salts, which exhibited a finely granular or occasionally radiating, fibrillated appearance, especially at the periphery (Figure 2B). Meningothelial whorls, psammoma bodies, or a distinct cartilaginous matrix were not found. The spaces between the round nodules were occupied by proliferation of fibroblasts in association with collagen fibers and diffuse infiltration of lymphocytes, plasma cells, histiocytes, and multinucleated giant cells. Histiocytes were occasionally laden with hemosiderin granules. Multinucleated giant cells had vesicular nuclei with prominent nucleoli and closely resembled osteoclasts, but they were distributed within fibrous tissue and not located in reabsorption lacunae of bone trabeculae (Figure 2C). In the lesion obtained from the second operation, the myxochondroid matrix was decreased in amount and partly replaced by fibrous tissue (Figure 2D), and the number of osteoclast‐like giant cells was considerably increased.

**Figure 2:**
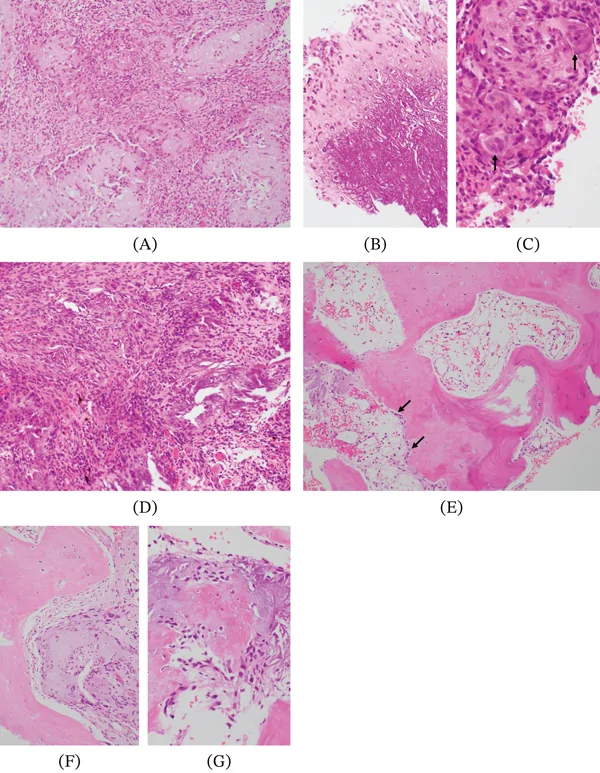
Histopathological findings of the CAPNON lesion. (A) The lesion consists of an aggregate of round nodules of the myxochondroid matrix rimmed by polygonal or short spindle cells. The intervening fibrous stroma contains many inflammatory cells. (B) The matrix shows deposition of basophilic calcium salts that exhibit a radiating, fibrillated appearance at the periphery. (C) Multinucleated giant cells resembling osteoclasts are found in the stroma (arrows). (D) In the regrowth lesion, the myxochondroid matrix was decreased in amount and partly replaced by fibrous connective tissue. (E) Irregularly anastomosing trabeculae of lamellar bone are formed. The surface of some bone trabeculae is partly lined by osteoblasts (arrows) (F) Some myxochondroid nodules are present within the bone marrow space. (G) The myxochondroid nodules occasionally merge into woven bone tissue. (A–G) Hematoxylin and eosin stain. (A,E–G) From the first operation. (B–D) From the second operation.

The second tissue component was lamellar bone tissue that formed irregularly anastomosing trabeculae. These bone trabeculae showed a peripheral osteoblastic lining in some places, and spaces corresponding to the bone marrow contained blood vessels and loose connective tissue, but no adipose tissue or hematopoietic cells (Figure 2E). Myxochondroid nodules were occasionally seen within the spaces corresponding to the bone marrow (Figure 2F). The lamellar bone tissue showed a gradual transition to immature woven bone, and the myxochondroid matrix merged into bone tissue in several places (Figure 2G). Neither atypical cells nor necrotic foci were found in the lesions obtained by both operations.

#### 2.2.2. Immunohistochemical Findings

Immunohistochemical studies were conducted using an automated immunostainer, Leica Bond‐Max (Leica Biosystems). Monoclonal or polyclonal primary antibodies against the following substances were used: S‐100 protein (polyclonal, Leica Biosystems, 1:500), glial fibrillary acidic protein (GFAP) (Clone EP672Y, Roche Diagnostics, prediluted), pancytokeratin (Clone AE1/AE3, Dako, 1:400), epithelial membrane antigen (EMA) (Clone E29, Dako, prediluted), podoplanin (Clone D2‐40, Dako, prediluted), epithelial cadherin (E‐cadherin) (Clone NCH‐38, Dako, 1:100), progesterone receptor (PgR) (Clone 1E2, Roche Diagnostics, prediluted), sex‐determining region Y‐box10 (SOX10) (Clone SP267, Ventana Medical Systems, prediluted), somatostatin receptor Type 2A (SSTR2A) (Clone EP149, Nichirei Bioscience, 1:4), CD34 (Clone QBEnd/10, Leica Biosystems, 1:400), CD68 (Clone KP1, Dako, prediluted), and CD163 (Clone 10D6, Novocastra, 1:200). Pretreatment by protease digestion or heat‐induced antigen retrieval was performed when indicated.

The majority of polygonal or short spindle cells rimming the myxochondroid nodules was intensely immunoreactive to SSTR2A (Figure 3A). These cells were also immunoreactive to EMA (Figure 3B) and podoplanin (Figure 3C) but negative to pancytokeratin, PgR, E‐cadherin, S‐100 protein, GFAP, SOX10, and CD34. Histiocytes infiltrating between the myxochondroid nodules were immunoreactive to CD68 or CD163, and osteoclast‐like, multinucleated giant cells showed intense immunoreactivity to CD68 (Figure 3D).

**Figure 3:**
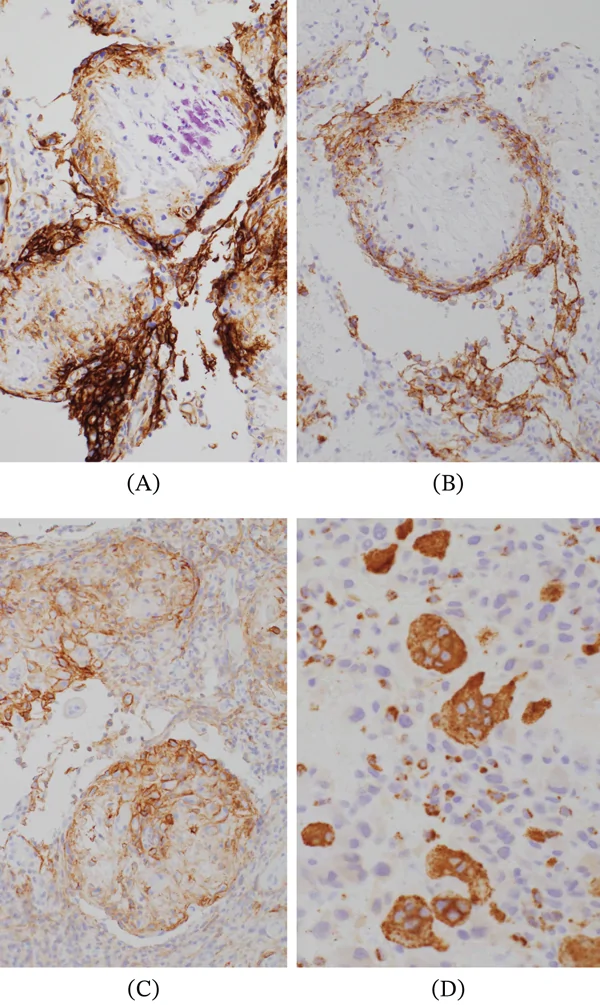
Immunohistochemical findings. Polygonal or short spindle cells rimming the myxochondroid nodules are intensely immunoreactive to (A) SSTR2A. These cells are also immunoreactive to (B) EMA and (C) podoplanin. Some cells within the nodules are also immunoreactive to these markers. (D) Osteoclast‐like, multinucleated giant cells are intensely immunoreactive to CD68. (A–D) From the second operation.

## 3. Discussion

CAPNON, a rare disorder that was first described by Rhodes and Davis [1], typically arises in the extra‐axial location of the brain and spinal cord and forms a mass lesion attached to the dura mater [1–4], but intraparenchymal lesions without dural attachment have also been reported [1, 3, 11–13]. According to a study on 37 cases by Ho et al. [4], lesions involved the spine or spinal cord in 76% of cases. Histopathologically, CAPNON is composed mainly of the myxochondroid matrix showing prominent deposition of calcium salts [2–4, 14]. The matrix is rimmed by polygonal or short spindle cells and forms “palisading granuloma”–like round nodules, and deposition of calcium salts forms a characteristic, radially arranged fibrillary structure at the periphery of the nodules [2–4, 14]. Formation of lamellar or woven bone tissue is another characteristic finding, and a rare case of adipocytic metaplasia has also been reported [12].

In the present case, the histopathological findings obtained from the second operation showed some features different from those in the primary lesion. The myxochondroid matrix was reduced in amount and partly replaced by fibrotic tissue, and number of osteoclast‐like giant cells was considerably increased. These changes might represent chronological alterations in the process of regressive changes of CAPNON lesions.

The pathological differential diagnosis of CAPNON includes various neoplasms, such as metaplastic (osteoblastic) meningioma [15], calcifying aponeurotic fibroma [16], and calcifying nerve sheath tumor. In the present case, the differentiation from metaplastic meningioma was important. The absence of solid, sheet‐like growth of meningothelial cells, formation of whorls, or psammomatous calcification excluded the diagnosis of meningioma. Peripheral nerve sheath tumors were also considered in the differential diagnosis because the tumor showed extension through the intervertebral foramina, and schwannomas with degenerative changes (so‐called “ancient schwannoma”) rarely exhibit deposition of calcium salts within the tumors. However, formation of the myxochondroid nodules or osteoclast‐like giant cells as seen in the present case is not seen in schwannomas. Some granulomatous or metabolic disorders such as tuberculosis, sarcoidosis, gout, and calcium pyrophosphate deposition disease [17], as well as necrobiotic lesions, such as seen in rheumatoid arthritis, should also be differentiated. In a few cases, coexistence of CAPNON with a neoplasm, such as meningioma [4, 13], ependymoma [11], dysembryoplastic neuroepithelial tumor [4, 18], and cerebral lipoma [14, 19], or nonneoplastic lesions, such as meningioangiomatosis [20] or neurenteric cyst [21], has been reported, and the possibility that CAPNON represents a reaction to these lesions has been discussed [11, 13, 18, 19, 21].

Polygonal or short spindle cells rimming the myxochondroid matrix are mostly immunoreactive to EMA [3, 4, 14, 18] and considered to be derived from meningothelial cells (arachnoid cells) [1, 18]. In the present case, these cells were demonstrated to also be immunoreactive to SSTR2A [22] and podoplanin [23]. The immunoreactivity to SSTR2A has also been reported by Soukup et al. [14].

The etiology and pathogenesis of CAPNON remain obscure, but it is considered most likely a reactive lesion to some unknown etiologic agents [3, 4]. In CAPNON involving the spine or spinal cord, concurrent degenerative diseases of the vertebral discs are frequently found [4], and the present patient also had lumbar disc herniation, although the levels of the CAPNON and disc herniation were different. Lu et al. [24] reported a unique case of the collision of CAPNON and rheumatoid nodules in the lumbar paravertebral region in a patient with systemic lupus erythematosus, and they discussed the involvement of an autoimmune‐mediated reaction in the pathogenesis of CAPNON. Whereas Ho et al. [4] stated that meningothelial cells were found in only 14% of CAPNON cases, in most cases of CAPNON, the lesions are attached to the dura mater [4], and many investigators have argued that meningothelial cells are involved in the pathogenesis of CAPNON [1, 3, 4, 18]. Also in the present case, meningothelial cells surrounded the myxochondroid matrix, and hyperplasia of meningothelial cells was supposed to have contributed to the pathogenesis of CAPNON lesions. Ichimata et al. [21] reported a case of CAPNON at the base of the frontal lobe that was associated with a neurenteric cyst and meningothelial hyperplasia adjacent to the CAPNON lesion. CAPNON is considered to be most likely a lesion of a dystrophic nature that is frequently accompanied by meningothelial hyperplasia [25].

The histopathological appearance, especially the pattern of calcification, in CAPNON lesions closely resembles that seen in tumoral calcinosis of somatic soft tissue, which is a disorder characterized by deposition of calcium hydroxyapatite and preferentially involves periarticular soft tissue [7–10]. The lesions are characterized by a central mass of the amorphous or granular calcified matrix that is bordered by florid proliferation of fibroblasts and chronic inflammatory cell infiltration [7–10]. Osteoclast‐like giant cells are also frequently found [7, 9]. The close histopathological resemblance of tumoral calcinosis to CAPNON suggests that these two disorders might share a common pathogenetic process, as some investigators have already pointed out [2, 3, 26].

The majority of cases of tumoral calcinosis belong to the primary or idiopathic type, and as etiologies of the secondary tumoral calcinosis, hereditary diathesis, long‐term hemodialysis, antecedent minor traumas, and metabolic disturbances such as hyperphosphatemia are presumed [7–10]. A disordered or exaggerated reparative reaction is proposed as a pathogenetic mechanism [7, 9]. On the other hand, in CAPNON, especially that arising in the spine or spinal cord, repetitive minor traumas or persistent mechanical overload because of movements of the vertebral column may be one of the inciting causes [4]. In the present case, the patient was a motorcycle mechanic and subjected to overload of the vertebral column by often lifting heavy objects in his daily work. He had a history of lumbar disc herniation. These situations might have operated as possibly contributing factors to his CAPNON.

In conclusion, we reported a case of thoracic intradural extramedullary CAPNON that showed postoperative regrowth. The CAPNON lesions accompanied reactive meningothelial hyperplasia surrounding myxochondroid nodules and showed chronological alterations of the histopathological features consisting of a decrease of the myxochondroid matrix and its replacement by fibrous connective tissue and a numerical increase of osteoclast‐like giant cells in the regrowth lesion.

## Funding

No funding was received for this manuscript.

## Ethics Statement

Kansai Medical University does not require ethics committee approval for a case report. This study was conducted in accordance with the Declaration of Helsinki.

## Consent

Written informed consent to use both clinical data and pathological materials was obtained from the patient.

## Conflicts of Interest

The authors declare no conflicts of interest.

## Data Availability

The data that support the findings of this study are available on request from the corresponding author. The data are not publicly available due to privacy or ethical restrictions.
